# Needlestick injuries among healthcare personnel in Qatar: A retrospective study

**DOI:** 10.5339/qmj.2021.35

**Published:** 2021-09-16

**Authors:** Shamja Sofia Razzakh, Muhammad Fazal Qureshi

**Affiliations:** Department of Medicine, Hamad Medical Corporation, Doha, Qatar E-mail: srazzakh1@hamad.qa

**Keywords:** needlestick injury, healthcare personnel, occupational hazard, post-exposure prophylaxis, Qatar

## Abstract

Background: A needlestick injury (NSI) is a serious occupational hazard among healthcare personnel (HCP), as it can cause transmission of blood-borne pathogens such as human immunodeficiency virus (HIV), hepatitis B virus (HBV), and hepatitis C virus (HCV). This study aimed to determine the frequency and distribution of reported NSIs, associated factors, use of post-exposure prophylaxis (PEP), and percentage of seroconversion among HCP in a major tertiary care hospital in Qatar.

Methods: This retrospective study analyzed NSIs among HCP reported in Hamad Medical Corporation facilities in Doha between May 01, 2017, and May 01, 2018. A surveillance follow-up period of 6 months commenced after the 1-year study period.

Results: A total of 130 NSIs were reported during the study period, with an overall incidence of eight injuries per 1000 HCP. The mean age was 34.6 ± 7.9 years. Among the reported cases, the proportion of female HCP (n = 72, 55.4%) was greater than that of male HCP (n = 58, 44.6%). Of 130 NSIs, 79 (60.8%) occurred in nurses, followed by 35 (26.9%) cases in doctors and 16 (12.3%) in other HCP. The total healthcare population comprised 49.6% of nurses and 18% of doctors. NSIs occurred in 10.1 per 1000 nurses and in 12.4 per 1000 doctors. Exposures mainly occurred in the operating theater, 35 (31.5%); inpatient wards, 24 (21.6%); and emergency department, 20 (18%). Common modes of injury were after use or before disposal of the device in 44 (44.4%) cases and during surgical interventions in 35 (35.4%) cases. Hollow-bore needles (64/98, 65.3%) were the most common devices involved. Source serology was documented in 71 (54.6%) cases of which 52 (73.2%) were normal, 9 (12.7%) were abnormal, and 10 (14.1%) were incomplete. Among the exposed HCP, 124 (95.4%) had adequate immunity to HBV. PEP for HBV was indicated in 6 (4.6%) and received by 4 (3.1%) HCP. NSI cases were followed up for 6 months post-exposure, and during this surveillance period, no seroconversion to HBV, HCV, or HIV was detected.

Conclusion: NSIs are common among HCP. In this study, most of the exposed HCP had adequate immunity to HBV. There was no hepatitis B, hepatitis C, or HIV transmission among the study cohort. Adherence to proper needle/sharps disposal techniques and safe practices during procedures will help prevent NSIs.

## Introduction

A needlestick injury (NSI) is one of the most significant occupational health issues among healthcare personnel (HCP).^1,2^ An NSI refers to a percutaneous exposure where the skin is penetrated by a needle that is potentially contaminated by blood or other bodily fluids.^3^ NSIs may occur during procedures such as administration of injections, recapping of needles, operative procedures, blood collection, intravenous line insertion, suturing, measurement of blood sugar, and improper sharps/needle disposal.^4^ The World Health Organization (WHO) estimates that more than two million occupational exposures to sharp injuries occur among 35 million HCP annually. According to the Centers for Disease Control and Prevention and European Agency for Safety and Health at Work reports, more than 385,000 and 1,000,000 NSIs occur annually among hospital HCP in the United States and Europe, respectively.^5^

An NSI is a serious occupational hazard in the transmission of blood-borne viruses such as hepatitis B virus (HBV), hepatitis C virus (HCV), and human immunodeficiency virus (HIV).^6^ The WHO has shown that, overall, 66,000 HBV, 16,000 HCV and 1,000 HIV infections occur annually worldwide among HCP due to their occupational exposure to percutaneous injuries.^5^ Prospective studies have estimated the transmission rates following percutaneous exposure to 6%–30% for HBV, 1.8% for HCV, and 0.3% for HIV.^7^ Among these, only HBV is preventable through immunization.^8^ If a patient is infectious, the specific risk of a single injury depends on several factors. Injuries with a hollow-bore needle, deep penetration, visible blood on the needle, a needle located in a deep artery or vein, or a needle with blood from terminally ill patients are known to increase the risk of HIV infection.^9^

In Qatar, there is no recent published data on NSIs.^10^ Given the shortage of national data, this study aimed to determine the frequency of reported NSIs and to explore its pattern and distribution across demographics, job category, location, mode of injury, and type of device involved. Furthermore, we analyzed data on the use of post-exposure prophylaxis (PEP) and the percentage of seroconversion to HIV, HBV, and HCV.

The study was conducted at Hamad General Hospital, the major tertiary care hospital under Hamad Medical Corporation (HMC) in Qatar, where HCP from all HMC facilities in Doha are to report in the event of a blood and body fluid exposure.

## Subjects And Methods

The study was a retrospective record-based data review of all NSIs among HCP in HMC facilities in Doha from May 01, 2017, to May 01, 2018. A surveillance follow-up period of 6 months commenced after the 1-year study period.

HMC facilities include the Hamad General Hospital, Women's Wellness and Research Center, Ambulatory Care Center, Qatar Rehabilitation Institute, Home Health Care Services, National Center for Cancer Care and Research, Heart Hospital, Communicable Disease Center, Rumailah Hospital, Dental Hospital, Mental Health Hospital, and Ambulance Service. During the study period, the total average number of HCP in these facilities was 15,683.

The study also included injuries caused by needle instruments and other sharps injuries (e.g., glass and surgical instruments). The study sample included all HCP from the aforementioned HMC facilities who reported an NSI during the study period. The inclusion criteria consisted of being a full-time employee in HMC. Workers who were not directly employed by HMC were excluded (e.g., housekeeping staff).

Data including age, gender, nationality, job category, location, mode of injury (circumstances leading to NSIs), and type of device involved were analyzed. Baseline serology data for HIV, HBV, and HCV for the exposed HCP at the time of NSIs were collected from electronic health records. Source patient serology, where available, was recorded. Administration of PEP for HBV and HIV and the percentage of seroconversion to HIV, HBV, and HCV among the reported cases were also determined.

The Medical Research Center of HMC approved the study protocol (MRC-01-18-394). The research involved the collection and study of existing data, documents, and records. The investigators recorded de-identified information of subjects directly or through identifiers linked to the subjects.

## Statistical Analysis

Descriptive statistics was used to summarize and determine the sample characteristics and distribution of various parameters related to NSIs. The overall cumulative incidence of NSIs was calculated using the following formula: number of reported NSIs (n)/number of HCP in the study period ×  1000 HCP.

Normally distributed data and results were reported as mean and standard deviation (SD), and the remaining results were reported as median and interquartile range (IQR). Data were analyzed and compared using Chi-square and Fisher's exact statistical tests. A two-sided p value <  0.05 was considered significant. All statistical analyses were performed using statistical packages SPSS 23.0 (SPSS Inc. Chicago, IL).

## Results

**Figure 1.**
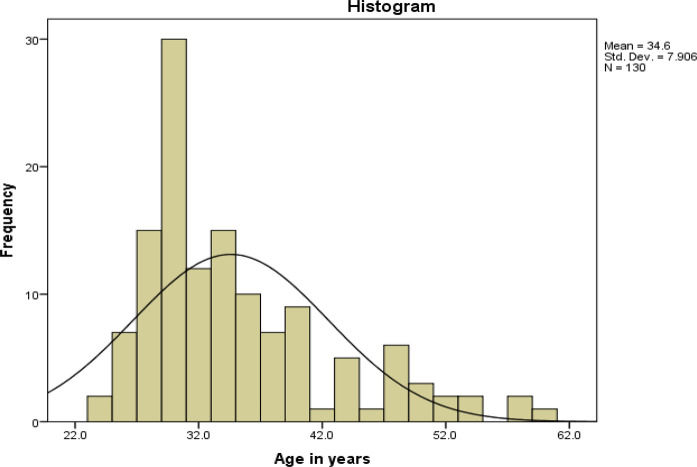
Age distribution histogram

A total of 130 NSIs were reported during the 1-year study period, with an overall incidence of eight injuries per 1000 HCP. The mean age of the exposed HCP was 34.6 ± 7.9 years. Among them, 106 (81.5%) were 21–40 years old (Figure 1). Of the reported cases, NSIs were higher among female HCP (n = 72, 55.4%) than among male HCP (n = 58, 44.6%). Most cases were reported by nurses (n = 79, 60.8%) (Table 1), followed by doctors (n = 35, 26.9%). Of the total HCP, 49.6% were nurses and 18% were doctors. NSIs occurred in 10.1 per 1000 nurses and in 12.4 per 1000 doctors. There were five paramedics, three anesthesia technicians, one each of lab, surgical, and dialysis technologists, as well as dental and pathology assistants, in the remaining HCP (n = 16, 12.3%) with NSI.

The operating theater (31.5%) was the most common site of NSI occurrence, followed by inpatient wards (21.6%), emergency department (18%), and intensive care units (8.1%). Other locations (10.8%) included the labor room, laboratory, outpatient, and ambulance.

The most frequent mode of injury was handling of needles after use and before disposal (44.4%), of which 18% was due to recapping of used needles. Surgical procedures accounted for 35.4% of NSIs, and the majority of these cases occurred while suturing (51%). Nonsurgical procedures contributed to 17.2% of the incidents, and 35% of these occurred during intravenous cannulation. Most cases involved hollow-bore needles (65.3%), followed by suture needles (19.4%) and scalpels (11.2%).

In 71 (54.6%) cases, source patient serology results were available, of which 52 (73.2%) had normal, 9 (12.7%) had abnormal, and 10 (14.1%) had incomplete serology (Table 2). The majority (95.4%) of the exposed HCP were immune to HBV. PEP for HBV was indicated only in 6 (4.6%) HCP, of which four received HBV vaccination. However, two were lost to follow-up after baseline serology. The reported cases were followed up for 6 months post-exposure, and during this surveillance period, any seroconversion to HBV, HCV, or HIV was not detected. Table 3 provides more details.

The distribution of gender, age, location, mode of injury, and type of instrument by occupational subgroups is shown in Table 4. The highest proportion of injuries among doctors occurred in the operating theater (n = 12, 41.4%). Most of the nurses sustained NSIs in hospital wards (n = 20, 30%) and emergency department (n = 19, 29%). NSIs mainly occurred during surgical procedures in doctors (n = 19, 63.3%). Most of the nurses (n = 33, 59%) sustain NSIs after use and before disposal of sharps. Differences in gender, mode of injury, and type of instrument among occupational subgroups were significant. However, owing to the minimal number of subjects in some subgroups, the significance might not be generalizable and not conclusive.

## Discussion

During the 1-year study period, 130 NSIs were reported. Overall, eight NSIs occurred per 1000 HCP. A proportion of injuries are likely not reported, which may underestimate the true incidence of NSIs. The level of under-reporting was not investigated in this study; however, it remains a significant problem among HCP worldwide. Several studies have estimated that the rate of under-reporting varies between 42% and 59%.^8,11–16^ Rates of NSI from studies using self-reported data are up to 10 times lower than those in prospective studies. Low perception of risk, embarrassment, fear of the consequences, and poor understanding of the reporting system are among the common reasons for not reporting NSI incidents by HCP.^8^

Most cases were reported in younger employees aged <  40 years. The average age of HCP was 36.8 years, and 55% of the overall workforce were <  40 years old. The lack of clinical experience is associated with higher NSI prevalence.^17^

In this study, 79 (60.8%) nurses have reported NSIs compared with 35 (26.9%) doctors among the 130 NSIs. Nurses constituted 49.6% of the total HCP, whereas doctors accounted for 18% of total HCP. In this study, 10.1 NSIs occurred per 1000 nurses and 12.4 occurred per 1000 doctors. Similar to our findings, studies from Malaysia and Singapore have found a higher NSI incidence among doctors than among nurses.^8,18^ In the UK, doctors and nurses almost equally contributed to the majority of NSIs over 10 years.^19^ However, several other studies have reported a higher rate of NSIs among nurses than among doctors.^14,20–25^ These discrepancies are likely due to different study designs; in the present study, we have estimated the incidence based on HCP subgroups.

During the study period, most of the NSIs occurred in the operating theater (31.5%), followed by inpatient wards (21.6%). There were comparatively fewer cases reported from the emergency department (18%) and intensive care unit (8.1%). The hospital ward was the most common site of occurrence in other studies.^8,16,20,21,23^

Improper disposal of needles accounted for 44% of the incidents, including recapping of used needles (18%). Surgical procedures were the second most common mode of injury (35%), and the majority of these injuries occurred during suturing (51%). These findings highlight the importance of adhering to proper sharps disposal techniques and safe practices during procedures.^8,16,20,21,26,27^ In line with other international studies, hollow-bore needles (65.3%) were the most common device involved.^8,21,28,29^ The healthcare workforce at HMC has diverse ethnicity, and the study did not find any cultural characteristic that has an implication on the results unlike other regional studies.^30^

In the present study, source baseline serology was known in 71 (54.6%) cases, of which 52 (73.2%) were normal. Moreover, 5.6% were hepatitis B surface antigen (HBsAg) positive, and 7% had anti-HCV positivity. None of the source patients tested positive for HIV. Availability of source baseline immunology status to HIV, HBV, and HCV facilitates management including PEP and further surveillance of the exposed HCP. It is essential to document the source to trace the baseline serology results. This will help prevent unnecessary anxiety for the HCP and help limit further follow-up testing that would otherwise be required. At HMC, the clinical policy on “management of occupational exposure to blood and body fluids” provides a standardized approach to reporting and managing NSIs. Moreover, HCP undergo mandatory periodic training on infection prevention and control practices.

None of the injured HCP tested positive for HIV, HBV, or HCV at baseline. Among the 130 HCP with exposure, 124 (95.4%) had adequate immunity to HBV. This rate is higher than those in other studies where vaccination coverage ranged from 68.3% to 88.4%.^20,23,31–33^ Vaccination against HBV is one of the best means to protect oneself from hepatitis B infection, and this is compulsory for HCP in HMC.

PEP was indicated for HBV only in 6 of 130 cases. There was no indication of HIV PEP for occupational exposure during the study period. There was no HBV, HCV, and HIV transmission in exposed HCP, consistent with other studies.^23,28^

Our data were limited to self-reported cases by HCP. Given the potential under-reporting of NSIs, obtained data may not represent all NSIs that occurred within the timeframe of the study. Furthermore, we do not have the exact gender distribution for the overall HCP population.

## Conclusion

NSIs are a major occupational challenge among HCP. Adherence to safe practice while handling sharps and proper disposal techniques will reduce and prevent this occupational hazard. In this study, most of the exposed HCP had adequate immunity to HBV. There was no transmission of blood-borne pathogens in the HCP during the post-exposure surveillance. Timely reporting and availability of baseline immunity status for the source, as well as affected HCP, is pivotal in the management of NSIs. This can be possible by the implementation of a uniform clinical policy across all healthcare facilities in Qatar.

### Statement of Ethics

The authors have no ethical conflicts to disclose. The Medical Research Center approved the study protocol (MRC-01-18-394). This study is exempt under MOPH guidelines. This is a Category 3 research involving the collection or study of existing data, documents, records, and information recorded by the investigator in such a manner that subjects cannot be identified, directly or through identifiers linked to the subjects.

### Disclosure

The authors have no conflicts of interest to declare.

### Funding

None.

## Figures and Tables

**Table 1:** Demographic and work-related factors associated with NSI

Variables	n	%

Gender		

Male	58	44.6

Female	72	55.4

Age group in years		

21–30	53	40.8

31–40	53	40.8

41–50	17	13.1

≥ 51	7	5.4

Mean age ± SD in years 34.6 ± 7.9		

Job category		

Doctors	35	26.9

Nurses	79	60.8

Others	16	12.3

Location of injury (n = 111)		

Ward	24	21.6

Operating theater	35	31.5

Intensive care unit	9	8.1

Emergency department	20	18.0

Other	23	20.7

Mode of injury (n = 99)		

Surgical procedures	35	35.4

Nonsurgical procedures	17	17.2

After use and before disposal of sharps	44	44.4

Others	3	3.0

Type of instrument involved (n = 98)		

Suture needle	19	19.4

Hollow-bore needle	64	65.3

Scalpel blade	11	11.2

Others	4	4.1


NSI: needlestick injury

**Table 2:** Serologic characteristics of source patients

Source patient serology	n	%

Documented	71	54.6

Normal	52	73.2

Abnormal	9	12.7

HBsAg positive	4	5.64

Anti-HCV positive	5	7.05

Anti-HIV positive	0	0

Incomplete serology	10	14.1

No Anti-HCV result	5	7.05

No Anti-HIV result	5	7.05


HBsAg: hepatitis B surface antigen; HIV: human immunodeficiency virus; HCV: hepatitis C virus

**Table 3:** HCP serology, PEP, and seroconversion

Variables	n	%

HCP baseline serology		

Normal	123	94.6

Not immune to HBV (anti-HBs < 10)	6	4.6

Anti-HCV positive	0	0

Anti-HIV positive	0	0

Incomplete serology (No anti-HBs at baseline)	1	0.8

PEP		

Indicated for HBV	6	4.6

Received	4	3.1

Not received	2	1.5

Indicated for HIV	0	0

Seroconversion		

Hepatitis B		

Yes	0	0

No	128	98.5

Unknown	2	1.5

Hepatitis C		

Yes	0	0

No	102	78.5

Unknown	28	21.5

HIV		

Yes	0	0

No	109	83.8

Unknown	21	16.2


HCP: healthcare personnel; PEP: post-exposure prophylaxis; Anti-HBs: hepatitis B surface antibody; HIV: human immunodeficiency virus; HCV: hepatitis C virus; HBV: hepatitis B virus

**Table 4:** Gender, age, location, mode of injury, and type of instrument by professional subgroups

Variable	Doctors (n = 35) n (%)	Nurses (n = 79) n (%)	Others (n = 16) n (%)	p-value

Gender				0.0001

Male	26 (74.3)	22 (27.8)	10 (62.5)	

Female	9 (25.7)	57 (72.2)	6 (37.5)	

Age in years				0.987

21–30	15 (42.9)	33 (41.8)	5 (31.3)	

31–40	14 (40)	32 (40.5)	7 (43.8)	

41–50	4 (11.4)	10 (12.7)	3 (18.8)	

≥ 51	2 (5.7)	4 (5.1)	1 (6.3)	

Location				0.006

Ward	3 (10.3)	20 (30.3)	1 (6.3)	

Operating theater	12 (41.4)	19 (28.8)	4 (25)	

Intensive care unit	2 (6.9)	6 (9.1)	1 (6.3)	

Emergency department	8 (27.6)	11 (16.7)	1 (6.3)	

Others	4 (13.8)	10 (15.2)	9 (56.3)	

Mode of injury				0.001

Surgical procedures	19 (63.3)	14 (25)	2 (15.4)	

Nonsurgical procedures	7 (23.3)	7 (12.5)	3 (23.1)	

After use and before disposal of sharps	4 (13.3)	33 (58.9)	7 (53.8)	

Others	0 (0)	2 (3.6)	1 (7.7)	

Type of instrument				0.001

Suture needle	11 (44)	6 (10)	2 (15.4)	

Hollow-bore needle	7 (28)	49 (81.6)	8 (61.6)	

Scalpel blade	5 (20)	4 (6.7)	2 (15.4)	

Others	2 (8)	1 (1.7)	1 (7.7)	


Some data such as location, mode of injury, and type of instrument were not documented; thus, “n” in each professional subgroup for these variables may not be complete. All respective rates were computed based on non-missing values.
